# Rice

**Published:** 1871-02

**Authors:** 


### RICE.

Boiled rice is among the most nutritious of all foods, and is the most easily digested, and, being cheap in all countries, it is the principal food of more than half of the human family, and is especially suitable for cold weather ; for one pound of it gives as much warmth to the body as four pounds of roast beef, and it is digested in about one fourth the time, that is, in one hour ; at the same time it does not give one quarter the power to work as roast beef. It is more universally used among the Chinese than any other nation, and is on that account, perhaps, prepared most philosophically ; they boil it thus : " Take a clean stew-pan with a close fitting top, then take a clean piece of white muslin, large enough to cover over the top of the pan, and hang down inside nearly to, but not in contact with the bottom. Into the sack so formed place the rice, pour over two cupfuls of water, and put on the top of the stew-pan, so as to hold up the muslin bag inside, and fit tight all around. Place the pan over a slow fire, and the steam generated from the water will cook the rice. Each grain, it is stated, will come out of the boiler as dry and as distinct as if just taken from the hull. More water may be poured into the pan, if necessary, but only sufficient to keep up the steam till the rice is cooked. The pan must not be heated so hot as to cause the steam to blow off the lid."

It ought to be extensively known that ordinary boiled rice, eaten with boiled milk, is one of the best remedies known for any form of loose bowels. Its efficacy is increased if it is browned like coffee, and then boiled and eaten at intervals of four hours, taking no other food or liquid whatever; its curative virtue is intensified if no milk is taken with it, and the patient will keep quiet in a warm bed; then it becomes an almost infallible remedy.

